# Complement in Reproductive White Adipose Tissue Characterizes the Obese Preeclamptic-Like BPH/5 Mouse Prior to and During Pregnancy

**DOI:** 10.3390/biology9090304

**Published:** 2020-09-22

**Authors:** Kelsey N. Olson, Dorien Reijnders, Viviane C. L. Gomes, R. Caitlin Hebert, Chin-Chi Liu, Jacqueline M. Stephens, Leanne M. Redman, Nataki C. Douglas, Jennifer L. Sones

**Affiliations:** 1 Veterinary Clinical Sciences, School of Veterinary Medicine, Louisiana State University, Baton Rouge, LA 70803, USA; kelsey.olson@pbrc.edu (K.N.O.); dorienreijnders88@gmail.com (D.R.); gomes3@lsu.edu (V.C.L.G.); cliu@lsu.edu (C.-C.L.); 2 Reproductive Endocrinology Laboratory, Louisiana State University-Pennington Biomedical Research Center, Baton Rouge, LA 70808, USA; caitlin.hebert@pbrc.edu (R.C.H.); leanne.redman@pbrc.edu (L.M.R.); 3 Adipocyte Biology Laboratory, Louisiana State University-Pennington Biomedical Research Center, Baton Rouge, LA 70808, USA; jsteph1@lsu.edu; 4 Division of Reproductive Endocrinology and Infertility, Department of Obstetrics, Gynecology and Women’s Health, Rutgers Biomedical and Health Sciences, Newark, NJ 07103, USA; nd537@njms.rutgers.edu

**Keywords:** preeclampsia, placenta, complement, obesity, adiposity

## Abstract

**Simple Summary:**

Preeclampsia is a life-threatening disorder that occurs in 10% of pregnant women worldwide. It presents as high blood pressure and multi-organ damage that when left untreated can result in death to both mother and baby. Treatment involves delivery of the placenta as well as the baby, often prematurely. Unfortunately, the cause of preeclampsia is unknown. However, there are known risk factors in women that may contribute to preeclampsia, including obesity. Increased adipose tissue (fat) has the potential to promote inflammation during pregnancy, which may negatively impact development of the baby and placenta, and lead to preeclampsia. This study aimed to show that reversal of maternal obesity through diet lowers inflammation in the fat and improves placental development, both of which have been shown to be necessary for pregnancy success. Utilizing obese female mice that demonstrate signs of preeclampsia (BPH/5), we showed that calorie restriction lowered inflammatory immune factors (complement) in the fat and restored essential growth factors in the developing placenta. In conclusion, these data suggest that targeting maternal obesity using calorie restriction and weight loss may improve pregnancy outcomes. Further studies are necessary to determine the influence of fat reduction in women and their likelihood of developing preeclampsia.

**Abstract:**

Preeclampsia (PE) is a serious hypertensive disorder of pregnancy characterized by abnormal placental development with an unknown etiology. To better understand which women will develop PE, a number of maternal risk factors have been identified, including obesity. Visceral white adipose tissue (WAT) contains inflammatory mediators that may contribute to PE. To explore this, we utilized the blood pressure high (BPH)/5 mouse model of superimposed PE that spontaneously recapitulates the maternal PE syndrome. We hypothesized that BPH/5 visceral WAT adjacent to the female reproductive tract (reproductive WAT) is a source of complement factors that contribute to the inflammatory milieu and angiogenic imbalance at the maternal–fetal interface in this model and in preeclamptic women. To test our hypothesis, we calorie-restricted BPH/5 females for two weeks prior to pregnancy and the first seven days of pregnancy, which attenuated complement component 3 (C3) but not complement factor B, nor complement factor D, (adipsin) in the reproductive WAT or the implantation site in BPH/5. Furthermore, calorie restriction during pregnancy restored vascular endothelial and placental growth factor mRNA levels in the BPH/5 implantation site. These data show maternal reproductive WAT may be a source of increased C3 during pregnancy, which is increased at the maternal–fetal interface in preeclamptic BPH/5 mice. It also suggests that calorie restriction could regulate inflammatory mediators thought to contribute to placental dysfunction in PE. Future studies are necessary to examine the effect of calorie restriction on C3 throughout pregnancy and the role of maternal obesity in PE.

## 1. Introduction

Approximately one in every three women of reproductive age (15–49 years) in the US has obesity; a risk factor for multiple adverse pregnancy outcomes, including gestational diabetes, hypertension, macrosomia, and preeclampsia (PE) [1] PE is a serious disorder of pregnancy characterized by high blood pressure and multiorgan dysfunction during the second half of gestation [2,3,4]. Although the etiology remains unknown, delivery of both the baby and placenta is often necessary to prevent fetal and maternal morbidity and mortality [1]. Therefore, the placenta is thought to play a causal role in PE [2].

Successful placental development depends on several tightly regulated physiological processes, including implantation, decidualization, and angiogenesis [2]. PE is associated with inadequate trophoblast invasion and remodeling of spiral arteries as a result of angiogenic, immune, and inflammatory dysregulation at the maternal–fetal interface [3,5]. Vascular endothelial growth factor (VEGF) and placental growth factor (PlGF) are necessary for placental vasculature development and are dysregulated in PE [6]. This, along with increased activation of the complement system, is associated with a systemic and placental angiogenic imbalance in preeclamptic women and rodent models of PE [7]. Furthermore, the complement system was previously indicated to be a key regulator of the maternal immune response during pregnancy. Failure of the complement system to properly mediate the clearance of waste and debris created during placentation may contribute to the pathogenesis of PE [8].

The complement system can be activated both systemically and locally through three distinct, well-described pathways, namely, classical, lectin, and alternative [3,9,10]. C3 (Complement component 3), a protein that plays a major role in the complement system, is the converging point of all three pathways, with the classical and alternative pathways mechanisms of great interest in PE. Both CfB (Complement factor B) and adipsin (Complement factor D), which are involved in the alternative complement pathway, contribute to the production of C3 [4,9]. Increased circulating C3a, a product of the proteolytic cleavage of C3, combined with obesity was associated with a high incidence of PE [4]. Regulation of C3 was demonstrated to be critical in ensuring a healthy pregnancy [8]. Furthermore, adipsin was suggested as a potential biomarker for susceptibility of PE in early pregnancy [11]. These observed increases in levels of circulating complement factors associated with PE and their link to obesity warrant further investigation.

Adipose tissue is a source of adipokines, cytokines, and complement proteins that, when activated, contribute to obesity-driven systemic inflammation [12]. It was proposed that visceral adipose tissue located adjacent to the developing placenta may influence angiogenic signaling at the maternal–fetal interface [3]. To investigate this phenomenon, we utilized the preeclamptic-like blood pressure high (BPH)/5 mouse model [2,13], which is used for studying pre-pregnancy and early pregnancy events that may contribute to PE, as BPH/5 spontaneously develops the cardinal maternal signs (late gestational hypertension and proteinuria) beginning at day 14 of pregnancy, which resolve upon delivery of pups and placentae [2,13]. BPH/5 females are hyperphagic and subsequently show increased adiposity, including increased reproductive visceral white adipose tissue (WAT) deposition adjacent to the reproductive tract before and during pregnancy [14]. Recently, Sones et al. demonstrated that expression of *VEGF* and *PlGF,* as well as *CfB* and *C3,* are upregulated at the maternal–fetal interface of BPH/5 mice at the peak of decidualization in murine pregnancy at embryonic day (e) 7.5 [7]. However, expression of complement C1q subcomponent subunit A (C1qa) was similar in BPH/5 and controls. Importantly, complement reduction by C3 convertase inhibition rescued BPH/5 pregnancies and was shown to improve fetoplacental development, including spiral artery remodeling and angiogenic imbalances in the placenta [15]. Furthermore, neutrophil infiltration and tumor necrosis factor (TNF)-α are also upregulated at the maternal–fetal interface in BPH/5 during early pregnancy, suggesting complement activation [15]. Additionally, DBA/2-mated CBA/J pregnancies demonstrate key features of PE including angiogenic dysfunction, proteinuria, and renal injury [16]. Complement inhibition during these pregnancies also showed improvements in maternal proteinuria and renal dysfunction [16]. This physiological evidence of complement regulation during early pregnancy in a model of superimposed PE supports further mechanistic investigations. We therefore hypothesize that reproductive WAT in female BPH/5 mice before and during pregnancy is a source of complement factors that contribute to the angiogenic imbalance at the maternal–fetal interface and that calorie restriction (CR) via pair feeding will attenuate this imbalance.

## 2. Materials and Methods

### 2.1. Animals

Virgin 8–12 week BPH/5 mice and control C57 (C57BL/6J) mice were used from in-house colonies maintained at the LSU School of Veterinary Medicine for all experiments. Mice were exposed to a standard 12 h light/dark cycle. Throughout all experiments, mice were fed ad libitum standard 5001 chow. Pair-feeding of nonpregnant BPH/5 female mice was done as previously described [17] by feeding the same amount of normal chow to BPH/5 as consumed by age-matched C57 female mice for 2 weeks. Intrastrain timed matings with day of vaginal plug detection denoted as e0.5 were performed with BPH/5 pair-feeding, beginning at e0.5 and continuing for 7 days until the mice were sacrificed, as previously described [14]. Reproductive WAT and implantation sites (uterus and embryo) were collected and flash frozen from ad libitum-fed and pair-fed, nonpregnant and pregnant BPH/5 and C57 female mice. Litter size and pregnancy rates were not significantly altered in BPH/5 mice at e7.5 after CR (Figure S1). All animal procedures were reviewed and approved by Louisiana State University (LSU) Institutional Animal Care and Use Committee. Mouse studies met the standards set forth by the National Institutes of Health (NIH) guidelines on the care and use of animals, United States Department of Agriculture (USDA) regulations, and the American Veterinary Medical Association Panel on Euthanasia.

### 2.2. Gene Expression Analysis

Total RNA was isolated from reproductive WAT of BPH/5 and C57 mice prior to pregnancy, as well as reproductive WAT and implantation sites at e7.5. Tissue homogenization and cDNA synthesis were performed as previously described [14]. qRT-PCR was used to determine the amount of *C3* (region corresponding to the alpha chain), *CfB*, *adipsin*, *VEGF*, and *PlGF* mRNA (Table S1), with 25 ng cDNA per reaction in triplicate using SYBR green. Relative expression was quantified and expressed as fold change relative to 18s rRNA using the 2^-delta Ct method, as previously described [14].

### 2.3. Protein Analysis

Protein lysates were made from e7.5 reproductive WAT samples by homogenizing samples in RIPA buffer with proteinase inhibitor and quantifying using the Bradford assay. A total of 40 ng of protein was loaded and separated using 4–12% Bis–Tris Protein Gel (ThermoFisher NP0321, Waltham, MA, USA) under reducing conditions, then transferred onto a nitrocellulose membrane blocked in 5% *w*/*v* nonfat dry milk/phospahe buffered saline tween (PBST). Complement C3 polyclonal (rabbit) primary antibody (1:2500) was used to detect C3 α and β bands (ThermoFisher PA5-21349, Waltham, MA, USA) with goat anti-rabbit horseradish peroxidase (HRP)-conjugated secondary antibody (Jackson Immunoresearch 11-035-003, West Grove, PA, USA) and mouse anti-actin primary antibody (1:5000, Sigma Aldrich, St. Louis, MO, USA) with goat-anti-mouse HRP-conjugated secondary antibody (Santa Cruz Biotechnologies, Dallas, TX, USA) (Figure S3). Band intensity was measured and normalized to actin with ImageJ software (NIH, Bethesda, MD, USA) following previously published methods [2].

### 2.4. Statistical Analysis

Statistical analyses were performed using GraphPad Prism, Version 8.0. Data were presented as mean ± standard error of the mean (SEM). qRT-PCR were analyzed with *t*-test or one-way ANOVA followed by Tukey post-hoc comparisons. Western blot quantifications were analyzed with one-tailed t tests to BPH/5. Assumptions of these models (linearity, normality of residuals, homoscedasticity of residuals) and influential data points were assessed by examining standardized residual and quantile plots, and the normality of residual was confirmed using the Shapiro–Wilk test. Statistical significance was defined as *p* < 0.05.

## 3. Results

### 3.1. Complement Factor 3 mRNA is Upregulated in the Reproductive WAT of BPH/5 Females Prior to Pregnancy

**Figure 1:**
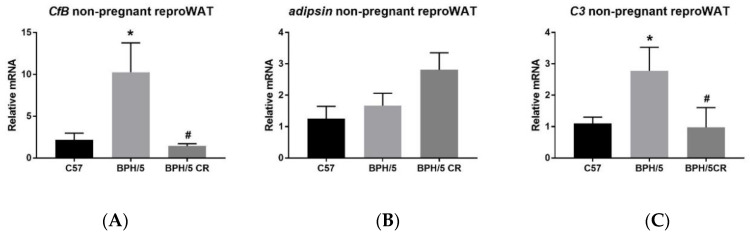
Complement factor B (CfB) and complement factor 3 (C3) mRNA is increased in female blood pressure high (BPH)/5 reproductive (repro) white adipose tissue (WAT) prior to pregnancy. (**A**) qRT-PCR analysis of complement factor B, (**B**) complement factor D (adipsin), and (**C**) complement factor 3 (C3) mRNA expression in reproductive (repro) WAT from nonpregnant, ad libitum-fed C57 and BPH/5, and calorie-restricted (CR) BPH/5 mice (*n* = 5–6, * *p* < 0.05 vs. C57, ^#^ *p* < 0.05 vs. BPH/5). Data are expressed as mean ± standard error of the mean (SEM).

We previously showed that CR via pair feeding not only reduces body weight and visceral WAT mass, but also lowers TNFα and interleukin (IL)-6 mRNA in BPH/5 female reproductive WAT [17]. Although complement dysregulation was demonstrated as early as e5.5 in BPH/5 pregnancy, a pre-pregnancy contribution is not yet clear. Thus, we sought to determine whether known obesity-related complement factors were upregulated in BPH/5 reproductive WAT and could be attenuated through restricting caloric intake for two weeks prior to pregnancy. We hypothesized that pre-existing maternal obesity is a source of increased complement in the BPH/5 mouse model. We measured key complement components and factors (*CfB, adipsin,* and *C3*) by qRT-PCR in the visceral reproductive WAT of ad libitum-fed, nonpregnant C57 and BPH/5 female mice. Although *adipsin* mRNA levels were not significantly different in the reproductive WAT between ad libitum-fed, nonpregnant C57 and BPH/5 females (Figure 1B), *CfB* and C3 mRNA were both elevated as well as reduced after CR (Figure 1A,C). This CR paradigm was effective in reducing *C3* and *CfB* mRNA in BPH/5 reproductive WAT.

### 3.2. Complement Factor 3 Dysregulation in Reproductive WAT Observed in Early BPH/5 Pregnancy and Can Be Attenuated by Reducing Adiposity

Calorie restriction (CR) via pair-feeding BPH/5 female mice at conception was shown to lower body weight, adiposity, and circulating leptin by e7.5 [14]. Furthermore, CR attenuated proinflammatory markers in the reproductive WAT and implantation sites at e7.5 in BPH/5 [14]. To better understand the adipose-dependent inflammatory profile in BPH/5 observed in early pregnancy [14], we measured *CfB*, *adipsin*, and *C3* mRNA in the reproductive WAT at e7.5 in ad libitum-fed C57 and BPH/5 females as well as BPH/5 females that were calorie restricted via pair-feeding for the first seven days of pregnancy. While complement mRNA expression was not different between C57 and BPH/5 pregnant reproductive WAT (Figure S2A–C), *VEGF* mRNA increased 10-fold in BPH/5 e7.5 reproductive WAT, which was attenuated by seven days of CR in BPH/5 females (Figure S2D).

**Figure 2:**
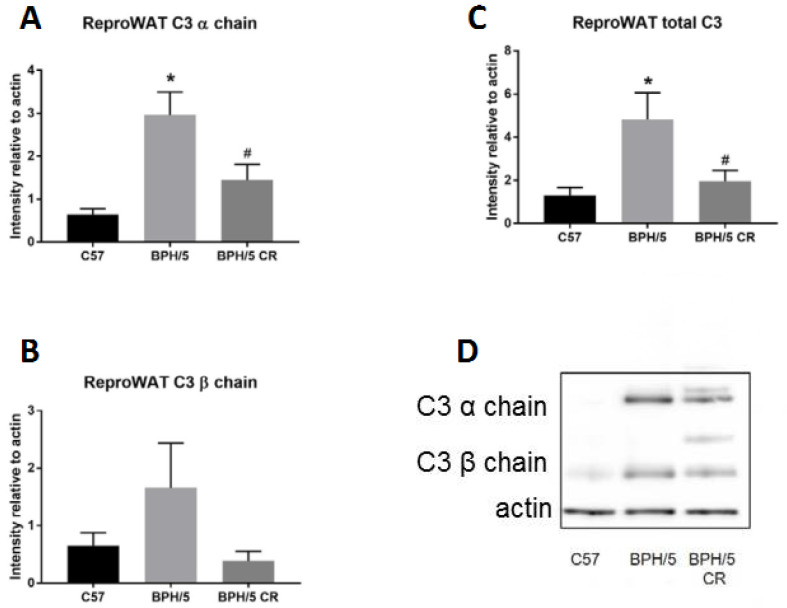
Complement factor 3 (C3) protein is increased in BPH/5 reproductive (repro) WAT in early pregnancy. (**A**) Quantification of C3 α chain, (**B**) C3 β chain, and (**C**) combined (α chain and β chain) C3 levels were measured in reproductive WAT of ad libitum-fed C57, ad libitum-fed BPH/5, and calorie-restricted (CR) BPH/5 mice at e7.5 (*n* = 3, * *p* < 0.05 vs. C57, ^#^ *p* < 0.05 vs. BPH/5). Data are expressed as mean ± SEM. (**D**) Representative Western blot gel of actin and C3 denatured protein levels.

Amelioration of several adverse pregnancy outcomes in BPH/5 occurred after C3 convertase inhibition [15], which decreases complement components downstream of CfB and adipsin, including C3 [18]. Therefore, further analysis of C3 was performed to better understand its expression in BPH/5 pregnancy, specifically C3 protein in the reproductive WAT at e7.5. C3 is cleaved to produce the two fragments C3a and C3b, while the constituent polypeptide chains that constitute the C3 protein are the α and β chains [19]. Of note, the C3 α and β chains are linked by disulfide bonds, which are reduced under Western blotting conditions and therefore appear as separate bands (Figure 2D) [20]. It was observed that C3 α chain and total C3 (α and β chains combined) protein levels were significantly increased in the reproductive WAT of BPH/5 females compared to ad libitum-fed C57 females at e7.5 (Figure 2A,C). BPH/5 females began CR at e0.5 to reduce adiposity, and both C3 α chain and total C3 levels were significantly reduced in reproductive WAT at e7.5 (Figure 2A,C). Individually measured, C3 β chain protein levels were not significantly changed in BPH/5 reproductive WAT at e7.5 after CR (*p* = 0.09, *n* = 3) (Figure 2B).

### 3.3. A Reduction in BPH/5 Maternal Adiposity Lowers C3 and Restores Angiogenic Balance at the Maternal–Fetal Interface in e7.5 BPH/5 Implantation Sites

**Figure 3:**
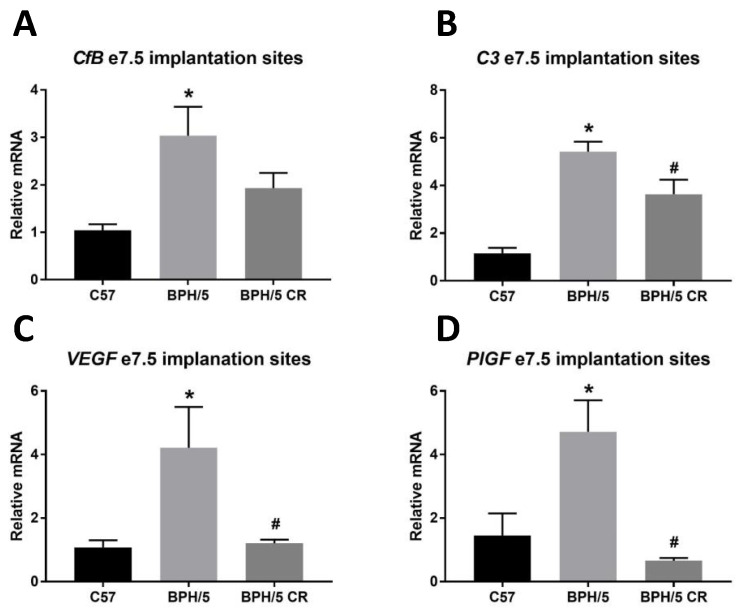
Complement factor 3 (C3), complement factor B (CfB), vascular endothelial growth factor (VEGF), and placental growth factor (PlGF) mRNA levels are increased in BPH/5 implantation sites in early pregnancy. (**A**) qRT-PCR analysis of complement factor B, (**B**) complement component 3 (C3), (**C**) vascular endothelial growth factor (VEGF), and (**D**) placental growth factor (PlGF) mRNA expression in implantation sites from e7.5 ad libitum-fed C57 and BPH/5, and calorie-restricted (CR) BPH/5 mice (*n* = 5–6, * *p* < 0.05 vs. C57, ^#^ *p* < 0.05 vs. BPH/5). Data are expressed as mean ± SEM.

An angiogenic imbalance in early pregnancy characterizes BPH/5 mice and other models of PE [1,7]. Complement inhibition was shown to restore placental VEGF levels in BPH/5 placenta [15]. To further investigate this, we sought to test whether complement reduction via CR improves angiogenic dysregulation in early BPH/5 pregnancy. We measured *CfB* and *C3,* as well as *VEGF* and *PlGF,* mRNA expression in e7.5 implantation sites. Consistent with previous findings [7], both complement (*CfB* and *C3*) and angiogenic factors (*VEFG* and *PlGF)* were upregulated in ad libitum-fed BPH/5 at e7.5 compared to ad libitum-fed C57 females (Figure 3A–D). *C3*, *VEGF* and *PlGF* were significantly reduced (Figure 3B–D) in BPH/5 CR females. However, *CfB* was unchanged in BPH/5 CR e7.5 implantation sites (Figure 3A).

## 4. Discussion

Maternal obesity with excessive visceral adipose tissue, which is rich in proinflammatory adipokines and complement proteins, may impair placentation and contribute to the development of PE in women with obesity and excess visceral adipose tissue [3]. We previously showed that adult, nonpregnant BPH/5 female mice exhibit increased body weight and proinflammatory reproductive WAT (i.e., TNF-α) compared to controls [17], which persists in early pregnancy (e7.5) [14]. At that time, the maternal–fetal interface in BPH/5 mice is characterized by an overexpression of inflammatory mediators, including *C3* and *CfB* but not *C1qa*, which is associated with an angiogenic imbalance [7]. We hypothesized that increased reproductive WAT could be a source of complement factors and, if reduced by maternal weight loss, would improve the angiogenic imbalance in BPH/5 implantation sites at e7.5, the peak of decidualization prior to placenta formation. The first aim of this study was to understand whether BPH/5 reproductive WAT before and in early pregnancy was a source of complement factors previously implicated in adverse PE outcomes. The second aim was to understand the effect of decreasing adiposity via reducing caloric intake on complement and angiogenic factor expression in BPH/5 pregnancies.

Our results showed that *C3* and *CfB*, but not *adipsin*, were increased in the reproductive WAT of nonpregnant BPH/5 females compared to controls, indicating that the reproductive WAT may be a source of complement components, but not necessarily exclusively those involved in the alternative complement pathway in the BPH/5 model. Pair-feeding was successful in reducing the expression of *C3* and *CfB* in the reproductive WAT of nonpregnant BPH/5 females, suggesting that reducing adiposity could be advantageous in correcting a dysregulated inflammatory reproductive WAT milieu prior to pregnancy. Furthermore, since maternal obesity is thought to contribute to the development of PE, we hypothesized that this dysregulation would continue into early pregnancy in BPH/5. As expected, C3 was increased in the reproductive WAT of BPH/5 females at e7.5, when *TNF-α* mRNA is also increased in BPH/5 reproductive WAT [14]. BPH/5 females, which were calorie restricted over the first seven days of pregnancy, presented significantly attenuated C3 levels in the reproductive WAT. As previously described, 25% CR over the first seven days of pregnancy is not thought to cause maternal undernutrition, and the amount of reproductive WAT in BPH/5 females by e7.5 is indeed significantly reduced [14]. Interestingly, CR did not reduce *TNF-α* in BPH/5 reproductive WAT, but it did attenuate *IL-6* [14]. This finding supported the idea that complement fragment 5a, which promotes the production of TNF-α [21], may not be activated in early BPH/5 pregnancy. The development of PE in human women was demonstrated to be associated with increased levels of C3 effector molecules, including C3a and other complement activation fragments in early gestation [4]. Therefore, further investigations are warranted in the BPH/5 model.

Complement activity is thought to contribute to PE by promoting an angiogenic imbalance localized at the maternal–fetal interface [3,22]. Our qRT-PCR data confirmed previously published RNA-Seq data, demonstrating that *C3* and *CfB* expression are upregulated in BPH/5 implantation sites at e7.5 [7]. The early pregnancy timepoint studied herein (e7.5) represents peak decidualization in mice, an important event for successful pregnancy. Thus, dysregulation during this important time could contribute to placental injury and later manifestation of PE. This study demonstrated that increased *C3* expression at the maternal–fetal interface was significantly attenuated in response to CR at this timepoint, although *CfB* was not. This discrepancy in *CfB* reduction in BPH/5 nonpregnant reproductive WAT, but not the e7.5 implantation site, may be due to the difference in CR timing with 14 days and 7 days imposed, respectively. Importantly, pregnancy rates and litter sizes at e7.5 were unchanged by CR in BPH/5 dams. This demonstrated that reduction of C3 did not have an adverse effect on early pregnancy events, such as fertilization and implantation rates, which was demonstrated in C3-deficient mice [23]. Furthermore, previously described *VEGF* and *PlGF* upregulation in BPH/5 e7.5 implantation sites confirmed the presence of an angiogenic imbalance during early pregnancy [7]. Overexpression of decidual VEGF was shown to promote elevations in the antiangiogenic factor soluble fms-like tyrosine kinase-1 (sFLT1) while producing a PE-like phenotype in a rodent model [24]. Hypoxia promotes increased expression of VEGF family members at the maternal–fetal interface [25]; therefore, it is interesting that the reproductive WAT adjacent to the implantation site at e7.5 in BPH/5 also showed increased *VEGF* mRNA. This finding is being further explored to determine individual cell expression patterns in the WAT fractions, i.e., stromal vascular cells, immune cells, and adipocytes, in this model and how they may impact local and systemic inflammation and angiogenic levels.

Prior research in BPH/5 mice showed that normalizing the expression of angiogenic factors at the maternal–fetal interface via the anti-inflammatory drug celecoxib was insufficient to correct the dysregulated complement expression observed [25]. This makes it especially notable that CR in early BPH/5 pregnancy attenuated *C3* expression at the maternal–fetal interface, as well as restored *VEGF* and *PlGF* expression levels to that of control mice. Although seven days of CR reduced *IL-6* mRNA in the BPH/5 e7.5 implantation site, it failed to alter TNF-α levels [14], suggesting that CR before pregnancy and continuing into early pregnancy would be more effective than CR initiated at the time of conception. The CR-induced maternal weight reduction further suggests that adipose tissue and, specifically, visceral depots may contribute to the inflammatory profile and concomitant placental angiogenic imbalances seen in PE. The relationship between the complement system and angiogenic dysregulation at the maternal–fetal interface was previously linked to immune cells, particularly macrophages [3]. It was suggested that proteins of the complement system polarize macrophages to produce antiangiogenic factors, thereby contributing to angiogenic imbalance [21]. Further research beyond the scope of this brief study is necessary to elucidate this relationship.

This study has some limitations. Calorie restriction during pregnancy is well described as a developmental origin of disease [26]. However, the paradigm of pair-feeding used in this study previously normalized body weight and adiposity prior to pregnancy in BPH/5 adult females; therefore, we do not think that this would cause undernutrition in the experimental conditions [17]. Additionally, the implementation of a 30% calorie restriction in pregnant women with obesity, intended to minimize gestational weight gain, was not reported to be associated with detrimental effects to mother or baby [27]. However, future research is necessary to understand the long-term consequences of this calorie restriction paradigm on BPH/5 offspring, including the effects on blood pressure and renal function. Additionally, BPH/5 mice may consume the allotted food within the pair-feeding paradigm in a way that could result in a fasted state. The effects of intermittent fasting are most prevalent when it occurs in the nocturnal phase, so pair-feeding took place in the morning to avoid introducing this as a variable [28]. Finally, complement proteins are synthesized throughout the body by a variety of cell and tissue types, including, but not limited, to liver tissue, neural tissue, immune cells, and adipose tissue [10]. We chose to focus on reproductive WAT due to its close proximity to the developing placenta. Additional research is required to better understand all sources of complement dysregulation that may contribute to PE in this model.

While maternal weight loss promotes pleiotrophic positive outcomes on the dam, the mechanistic studies herein provide new knowledge into the beneficial effects of adiposity reduction in pregnancy. Complement, adiposity, and angiogenic associations should be further investigated to identify causation in this model. Preliminary data in BPH/5 supports improvement in maternal and fetal adverse PE outcomes, however, studies are ongoing to investigate the contribution of obesity on the hypertensive phenotype in BPH/5 pregnant mothers. In summary, reduction of maternal adipose tissue during early pregnancy could ameliorate the aggravated inflammatory profile that characterizes BPH/5 reproductive WAT and implantation sites prior to placenta formation, which was shown to be causal in fetoplacental development and hypertension in this model.

## 5. Conclusions

**Figure 4:**
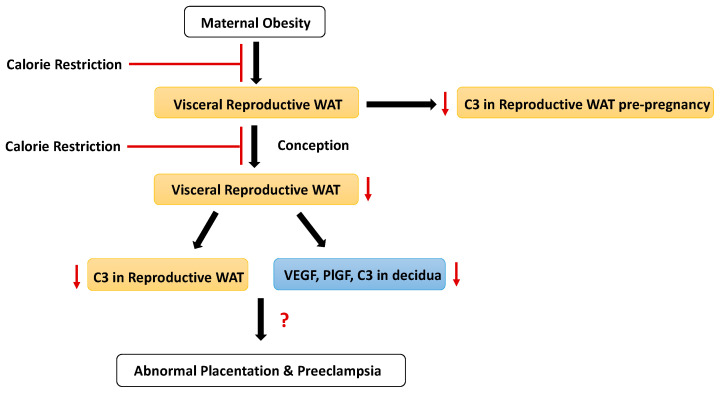
Working hypothesis: Maternal obesity results in increased visceral reproductive white adipose tissue (WAT) with increased levels of complement component 3 (C3) in BPH/5 females. Increases in reproductive WAT and C3 begins before pregnancy and is associated with misexpression of vascular endothelial growth factor (VEGF), placental growth factor (PlGF), and C3 in BPH/5 implantation sites at peak decidualization (e7.5), which may contribute to abnormal placentation and the development of preeclampsia. Calorie restriction to reduce adiposity either before or during early pregnancy may attenuate the C3 and angiogenic factor dysregulation seen in early pregnancy to improve pregnancy outcomes.

Maternal obesity prior to and during early pregnancy may be a therapeutic target for PE intervention. A reduction in adipose tissue by CR holds promise for ameliorating inflammatory and angiogenic dysregulation at the maternal–fetal interface, thereby reducing the risk of PE (Figure 4). Unfortunately, given the systemic effects of CR, it is difficult to elucidate the precise mechanism of action whereby reduced maternal adiposity improves pregnancy outcomes. Of note, this study was conducted in a mouse model to begin understanding the complex PE etiology; extrapolating interventions from animal models to humans requires more research. Future studies should inquire into the mechanisms linking obesity, the immune system, and PE, particularly regarding identification of the immune cell types responsible for producing complement components and contributing to the inflammatory milieu characterizing the maternal–fetal interface during PE.

## Supplementary Materials

The following are available online at https://www.mdpi.com/2079-7737/9/9/304/s1, Figure S1: Calorie restriction does not impact pregnancy rates not litter size in BPH/5 early pregnancy. (A) Percent of ad libitum fed C57BL/6 and BPH/5 as well as calorie restricted (CR) BPH/5 mice that become pregnant after timed mating (*n* = 4–9). (B) Measurement of litter size in ad libitum fed C57BL/6 and BPH/5 as well as CR BPH/5 mice (*n* = 8–15, * *p* < 0.05). Data are expressed as mean ± SEM, Figure S2: Vascular endothelial growth factor (VEGF) mRNA is increased in ad libitum fed BPH/5 reproductive (repro) WAT in early pregnancy. (A) qRT-PCR analysis of complement factor 3 (C3), (B) complement factor D (adipsin), (C) complement factor B (CfB) and (D) vascular endothelial growth factor (VEGF) mRNA expression in reproductive (repro) WAT from e7.5 pregnant ad libitum fed C57 and BPH/5, and calorie restricted (CR) BPH/5 mice (*n* = 5–6, * *p* < 0.05 vs C57, # *p* < 0.05 vs BPH/5 ad libitum). Data are expressed as mean ± SEM, Figure S3: Complement factor 3 (C3) protein is increased in BPH/5 reproductive (repro) WAT in early pregnancy. Annotated western blot gel of actin and C3 denatured (α and β chains) protein levels in repro WAT. *n* = 3 per group, Table S1 Forward and reverse primer sequences for complement component 3 (C3), complement factor B (CfB), complement factor D (adipsin), vascular endothelial growth factor (VEGF), and placental growth factor (PlGF). Primer sequences were obtained from published literature referenced above. These primer sequences were used in qRT-PCR experiments.
