# Supplementary material for: Complement in Reproductive White Adipose Tissue Characterizes the Obese Preeclamptic-Like BPH/5 Mouse Prior to and During Pregnancy
Source: Biology (Basel). 2020 Sep 22;9(9):304. doi: 10.3390/biology9090304 (PMC7564206; doi:10.3390/biology9090304)
Supplement: Supplementary file 1 [file biology-09-00304-s001.pdf]

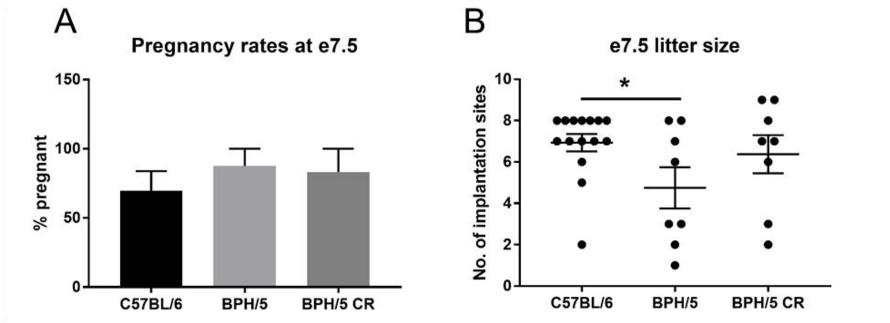


**Figure S1.** Calorie restriction does not impact pregnancy rates not litter size in BPH/5 early pregnancy. (**A**) Percent of ad libitum fed C57BL/6 and BPH/5 as well as calorie restricted (CR) BPH/5 mice that become pregnant after timed mating ($n = 4–9$). (**B**) Measurement of litter size in ad libitum fed C57BL/6 and BPH/5 as well as CR BPH/5 mice ($n = 8–15$, * $p < 0.05$). Data are expressed as mean ± SEM.

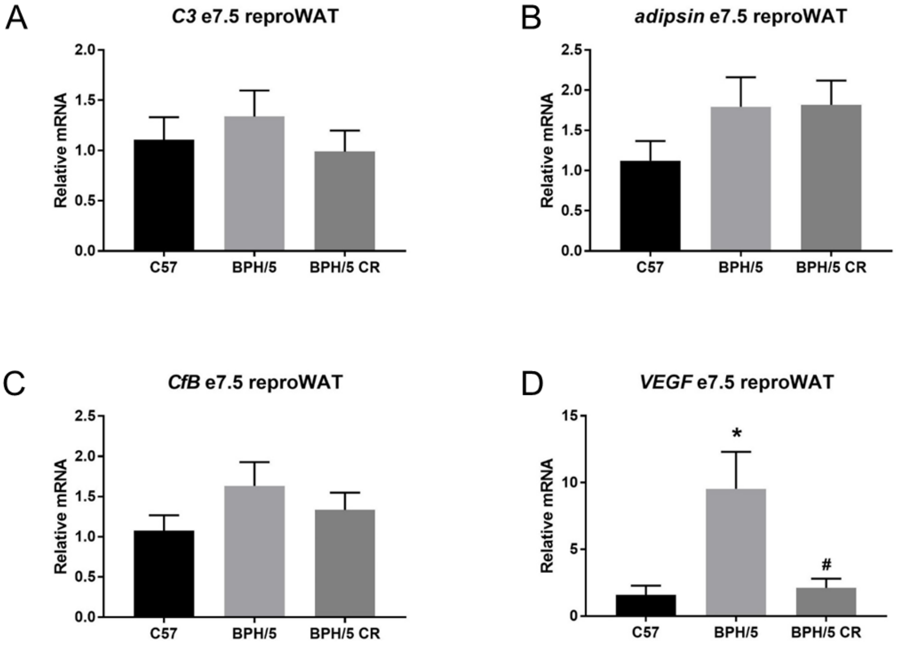


**Figure S2.** Vascular endothelial growth factor (VEGF) mRNA is increased in ad libitum fed BPH/5 reproductive (repro) WAT in early pregnancy. (**A**) qRT-PCR analysis of complement factor 3 (C3), (**B**) complement factor D (adipsin), (**C**) complement factor B (CfB) and (**D**) vascular endothelial growth factor (VEGF) mRNA expression in reproductive (repro) WAT from e7.5 pregnant ad libitum fed C57 and BPH/5, and calorie restricted (CR) BPH/5 mice ($n = 5–6$, * $p < 0.05$ vs C57, # $p < 0.05$ vs BPH/5 ad libitum). Data are expressed as mean ± SEM.

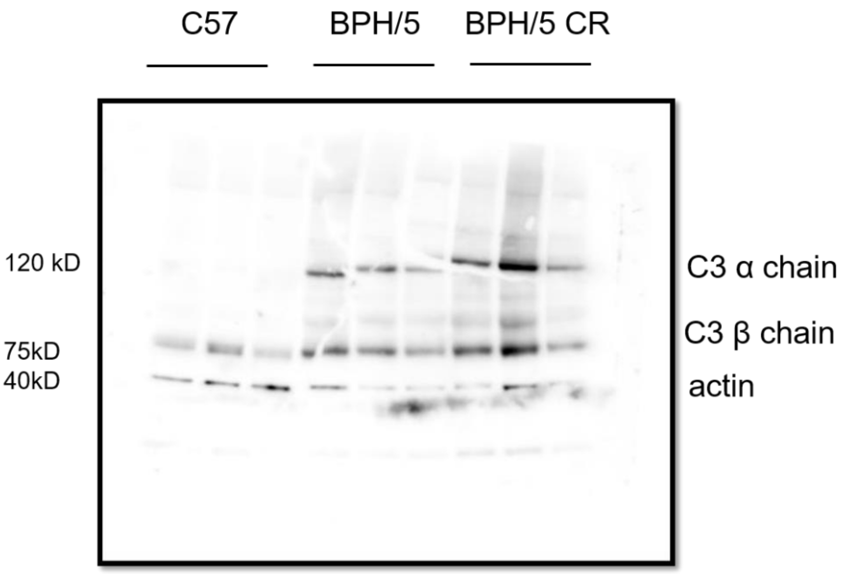


**Figure S3.** Complement factor 3 (C3) protein is increased in BPH/5 reproductive (repro) WAT in early pregnancy. Annotated western blot gel of actin and C3 denatured ($\alpha$ and $\beta$ chains) protein levels in repro WAT. $n = 3$ per group.

**Table S1.** Forward and reverse primer sequences for complement component 3 (C3), complement factor B (CfB), complement factor D (adipsin), vascular endothelial growth factor (VEGF), and placental growth factor (PlGF). Primer sequences were obtained from published literature referenced above. These primer sequences were used in qRT-PCR experiments.

| Gene | Forward Sequence | Reverse Sequence | Reference |
|---|---|---|---|
| *C3* | CACCGCCAAGAATCGCTAC | GATCAGGTGTTTCAGCCGC | [1] |
| *CfB* | GAAACCCTGTCACTGTCATTC | CCCCAAACACATACACATCC | [1] |
| *Adipsin* | GCTATCCCAGAATGCCTCGTT | CCACTTCTTTGTCCTCGATTGC | [2] |
| *VEGF* | CTTGTTCAGAGCGGAGAAAGC | CATCTGCAAGTACGTTCGTT | [3] |
| *PlGF* | TCTGCTGGGAACAACTCAACA | GTGAGACACCTCATCAGGGTAT | [3] |

## References


1. Sones, J.L.; Merriam, A.A.; Seffens, A.; Brown-Grant, D.-A.; Butler, S.D.; Zhao, A.M.; Xu, X.; Shawber, C.J.; Grenier, J.K.; Douglas, N.C. Angiogenic factor imbalance precedes complement deposition in placentae of the BPH/5 model of preeclampsia. *FASEB J.* **2018**, *32*, 2574–2586, doi:10.1096/fj.201701008r.
2. Searfoss, G.H.; Jordan, W.H.; Calligaro, D.O.; Galbreath, E.J.; Schirtzinger, L.M.; Berridge, B.R.; Gao, H.; Higgins, M.A.; May, P.C.; Ryan, T.P. Adipsin, a biomarker of gastrointestinal toxicity mediated by a functional gamma-secretase inhibitor. *J. Biol. Chem.* **2003**, *278*, 46107–46116.
3. Woods, A.K.; Hoffmann, D.S.; Weydert, C.J.; Butler, S.D.; Zhou, Y.; Sharma, R.V.; Davisson, R.L. Adenoviral delivery of VEGF121 early in pregnancy prevents spontaneous development of preeclampsia in BPH/5 mice. *Hypertension* **2010**, *57*, 94–102, doi:10.1161/HYPERTENSIONAHA.110.160242.
